# Spatiotemporal Dynamics in the Burden of Lip and Oral Cavity Cancer and Attributable Risk Factors in Asia (1990–2021)

**DOI:** 10.3390/healthcare13121377

**Published:** 2025-06-09

**Authors:** Dan Lin, Xinping Lu, Ri Ma, Xiaojuan Zeng

**Affiliations:** 1 Guangxi Key Laboratory of Oral and Maxillofacial Rehabilitation and Reconstruction, Guangxi Clinical Research Center for Craniofacial Deformity, College & Hospital of Stomatology, Guangxi Medical University, Nanning 530021, China; 202000129@sr.gxmu.edu.cn (D.L.);; 2 College of Stomatology, Guangxi Medical University, Nanning 530021, China

**Keywords:** Asia, lip and oral cancer, global burden of disease, age standardized DALYs, risk factors

## Abstract

**Background/Objectives:** Lip and oral cavity cancer (LOC) remains a critical public health challenge in Asia. This study evaluated spatiotemporal trends and risk factor contributions to LOC-related disability-adjusted life years (DALYs) from 1990 to 2021 to inform evidence-based healthcare policies. **Methods:** Using Global Burden of Disease (GBD) 2021 data, we analyzed LOC DALYs stratified by age, gender, risk factors (smoking, alcohol use, tobacco chewing), and subregions in Asia. Temporal trends were quantified via estimated annual percentage change (EAPC) across five geographic regions and sociodemographic index (SDI) categories. Age–period–cohort (APC) modeling was used to assess age-specific risk distributions. **Results:** From 1990 to 2021, Asia’s age-standardized DALY rate (ASDR) for LOC marginally increased (EAPC: 0.0883, 95% CI: 0.0802–0.0963). The alcohol-related ASDR increased sharply (EAPC: 1.033, 95% CI: 1.00–1.06), whereas decreases were detected in the smoking- and tobacco chewing-attributable ASDRs. Pronounced upward trends were observed in South Asia and low/low-middle-SDI regions. Tobacco chewing was the primary risk factor for women and for the populations in South Asia and lower-SDI regions, whereas smoking dominated among men and those in other geographic regions and in higher-SDI areas. APC analysis revealed age-driven increases in ASDR, with alcohol use and tobacco chewing risk increased with age. Notably, the steepest ASDR increase occurred in individuals aged 20–25 years. **Conclusions:** The LOC burden in Asia reflects divergent risk factor dynamics. Policy strategies must prioritize geographic and demographic targeting: alcohol control in rapidly developing areas and intensified tobacco cessation programs in endemic zones. Early prevention efforts focusing on adolescents and tailored to subregional risk profiles are essential to mitigate future disease burden.

## 1. Introduction

Based on the Global Burden of Disease (GBD) Study estimates, lip and oral cavity cancer (LOC) ranks 16th in global incidence and 15th in mortality among all cancers, accounting for a substantial portion of the global cancer burden [1]. LOC can involve the lower lip, the front two-thirds of the tongue, the floor of the mouth, and the alveolar bone of the upper and lower jaw [2], with the predominant form being squamous cell carcinoma [3]. LOC reduces patients’ quality of life to varying degrees and causes severe economic and psychological burdens [4], and is associated with a very low overall survival rate of 50–60%, as patients typically have stage III or IV disease at diagnosis. Despite recent therapeutic advances, the five-year survival rate of patients with late-diagnosed LOC has not improved [5,6].

The burden of LOC is a serious public health threat in Asia. The number of disability-adjusted life years (DALYs) for LOC increased from 1,723,076 in 1990 to 3,954,826 in 2019 [7], accounting for 72.06% of the total number worldwide. Moreover, changes in the population, gender ratio, age, social division of labor, and economic status are occurring in Asia, which has led to heterogeneity in the LOC disease burden [8]. Asians have distinct cultural practices and different patterns of tobacco and alcohol use, which are key risk factors that predispose them to oral cancer in a dose- and time-dependent manner [9]. Furthermore, many cases of oral cancer are caused by the combined effects of tobacco and alcohol, which are greater than the sum of their individual effects [10]. From 1990 to 2019, there were significant changes occurred in the oral cancer burden status across regions, with a more pronounced downward trend seen in high-income countries [11,12]. However, the evidence on the distribution and trends of these risk factors for oral cancer that correspond to different geographical and economic levels is still limited. Overall, the economic benefits of primary risk factor prevention far outweigh those of treatment. Therefore, an assessment of risk factors related to the disease burden of and regional and temporal heterogeneity in LOC in Asia is urgently needed.

DALYs consider the prevalence of a disease and its effects on mortality as well as morbidity and help to quantify and rank the disease burden of disease according to specific causes [13]. The age-standardized rate (ASR) is a metric that can greatly eliminate the impacts of differences in age structure [14] and can provide clues to changes in risk factors. These metrics collectively inform evidence-based policymaking, as demonstrated by the WHO Framework Convention on Tobacco Control (FCTC), which has been adopted by 182 parties since 2003, which reduced tobacco-related burdens through taxation, smoking bans, and advertising restrictions [12]. Aligned with the WHO Global Strategy for Oral Health (2023–2030), this study addresses Asia’s persistent epidemiological disparities in LOC burden. Leveraging 2021 Global Burden of Disease (GBD) data, we analyze age-standardized DALY rates (ASDRs) across Asian subregions, dissecting risk factor contributions by gender, age, and region. The findings provide nuanced, actionable insights for tailoring prevention strategies, to curb the escalating LOC burden, thus offering critical updates to guide policy refinement in high-risk areas.

## 2. Materials and Methods

### 2.1. Data Resources

We explored the data sources related to the burden of LOC through the Global Health Data Exchange (GHDx), an online data source query tool (http://ghdx.healthdata.org/ (accessed on 29 September 2024)) that was publicly released in May 2024. In the GBD study, LOC was defined according to the International Classification of Diseases, 10th revision, version 10 (ICD-10) codes C00–C08 [2]. We extracted data on the three main risk factors (smoking, alcohol use, and tobacco chewing) for DALYs according to the GBD study operational guidelines and considered disease type, age, gender, and region. According to the existing classification in the GBD database, Asian countries were divided into five regions: West Asia, East Asia, Southeast Asia, Central Asia, and South Asia. Specifically, we extracted data for North Africa and the Middle East as proxies for West Asia, as 17 of the 21 countries in the GBD database are in western Asia. These countries are geographically close to each other and have very similar customs and cultures [7]. Furthermore, we also utilized sociodemographic index (SDI) data from the GBD database as a reference for SDI levels. The SDI is an important indicator of social and economic development that is tightly correlated with health outcomes and is considered a good indicator for predicting and assessing disease burden [15,16]. Given the absence of direct SDI calculations for Asia in the GBD database, we obtained data on LOC in patients of both genders from 51 countries across five regions from 1990 to 2021.

### 2.2. Statistical Analysis

We leveraged population figures provided by the World Bank (https://data.worldbank.org.cn/indicator/SP.POP.TOTL (accessed on 13 October 2024)) to proportionally calculate risk-adjusted and ASDRs for lip and oral cavity cancer across various regions in Asia in a proportional manner. The natural logarithm of the ASR is linear with time, as shown in the following equation:

Y=α+βX+ε

Y represents ln (ASR), X represents the calendar year, ε represents the error/noise term, and β represents the slope, which indicates a linear positive or negative trend in the ASR.

The estimated annual percentage change (EAPC) is used as the annual percentage change in the ASDR over a given time interval and is used to assess trends in the burden of LOC. The EAPC was calculated via the following formula:

$$EAPC=100^{\prime }(exp(\beta )-1)$$

A linear model was used to obtain the formula for the EAPC and its 95% confidence interval. When both the lower limit of the confidence interval and the EAPC are positive, the ASR is considered to show an upward trend.

Age-period-cohort (APC) analysis was used to evaluate the trends of LOC according to the following age stratifications in the GBD 2021 study: 15–19, 20–24, 25–29, 30–34, 35–39, 40–44, 45–49, 50–54, 55–59, 60–64, 65–69, 70–74, 75–79, 80–84, 85–89, and 90–94 years. The age effect represents the different risks of outcomes associated with different ages [17]. Net drift and local drift were used in the APC model to show the overall time trend of the ASDR. The net drift represents the overall APC. Local drift represents the APC in the ASDR for a specific age group [18]. The Wald chi-square test was used to analyze statistical significance. All the statistical analyses were performed via R software (version 4.0.2). A *p* value of <0.05 was considered statistically significant.

## 3. Results

### 3.1. Burden of LOC in Asia

From 1990 to 2021, the DALYs for LOC increased from 1,781,211.87 in 1990 to 4,283,251.95 in 2021, with a cumulative increase of 129.18%. In Asia, the DALYs were 2.4 times higher in 2021 than in 1990 and accounted accounting for 72.91% of the global burden of oral cancer, while the ASDR showed a slight upward trend, with an EAPC of 0.09 (95% CI, 0.08 to 0.10) (Table 1 and Table S1).

**Figure 1:**
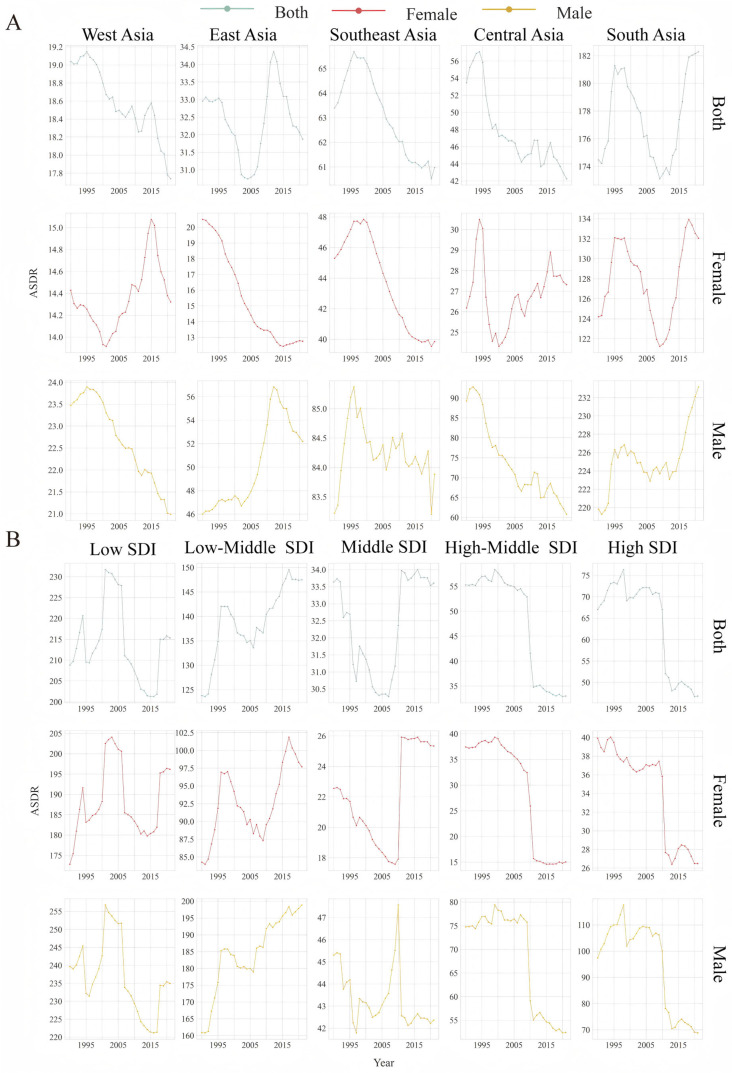
Trends in the ASDR from 1990 to 2021 by gender in Asia. (**A**) Geographical region. (**B**) SDI subregion. SDI: sociodemographic index; DALYs: disability-adjusted life years; ASDR: age-standardized DALY rate.

**Figure 2:**
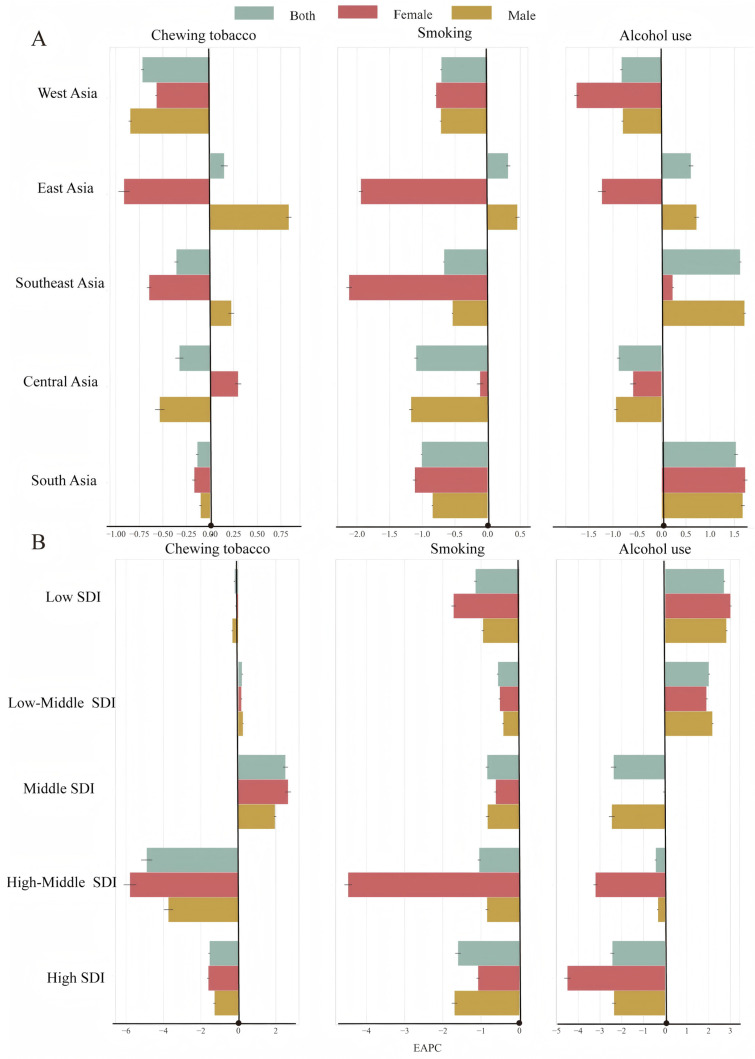
EAPCs in LOC attributable to each risk factor by gender across geographical and SDI regions. (**A**) Geographical regions. (**B**) SDI regions. SDI: sociodemographic index; EAPC: estimated annual percentage change; Origin: black solid dot (EAPC = 0, EAPC > 0 on the right of Y axis, EAPC < 0 on the left of Y axis).

From a regional and temporal perspective, since 1990, South Asia has consistently exhibited the highest ASDR, which showed an increasing trend, an EAPC of 0.02 (95% CI, 0.01 to 0.03) (Figure 1A, Table 1), and an absolute magnitude almost six times greater than that of East Asia in 2021 (Table S2). Moreover, the ASDR among men in South Asia continued to increase for almost the past 10 years (Figure 1A, Table S2). Additionally, the ASDR in East Asia has slightly increased over the last 31 years, with an EAPC of 0.01 (95% CI, −0.01 to 0.03). However, compared with West Asia and Southeast Asia, Central Asia showed a noticeable downward trend, as shown in Figure 2A and Table S3.

In terms of the SDI, the low-SDI regions clearly had the highest ASDR (215.36; 95% CI, 162.27 to 279.07), which was nearly five times greater than that of the high-SDI regions (46.81; 95% CI: 39.27 to 55.72) in 2021 in absolute terms (Figure 1B and Table S4). The low-middle-SDI and middle-SDI regions showed an increasing trend, with EAPCs of 0.37 (95% CI, 0.35 to 0.39) and 0.13 (95% CI, 0.10 to 0.15), respectively (Figure 1B and Table 1).

With respect to gender, in terms of both the geographical region and the SDI, the ASDR among males was, on average, approximately two times greater than that among females and showed a slight upward trend (males: EAPC, 0.17 (95% CI, 0.16 to 0.17); females: EAPC, −0.0370 (from −0.02 to −0.05) (Figure 2A,B and Table 1).

### 3.2. Trends in the ASDR for LOC Attributable to Alcohol Use

**Figure 3:**
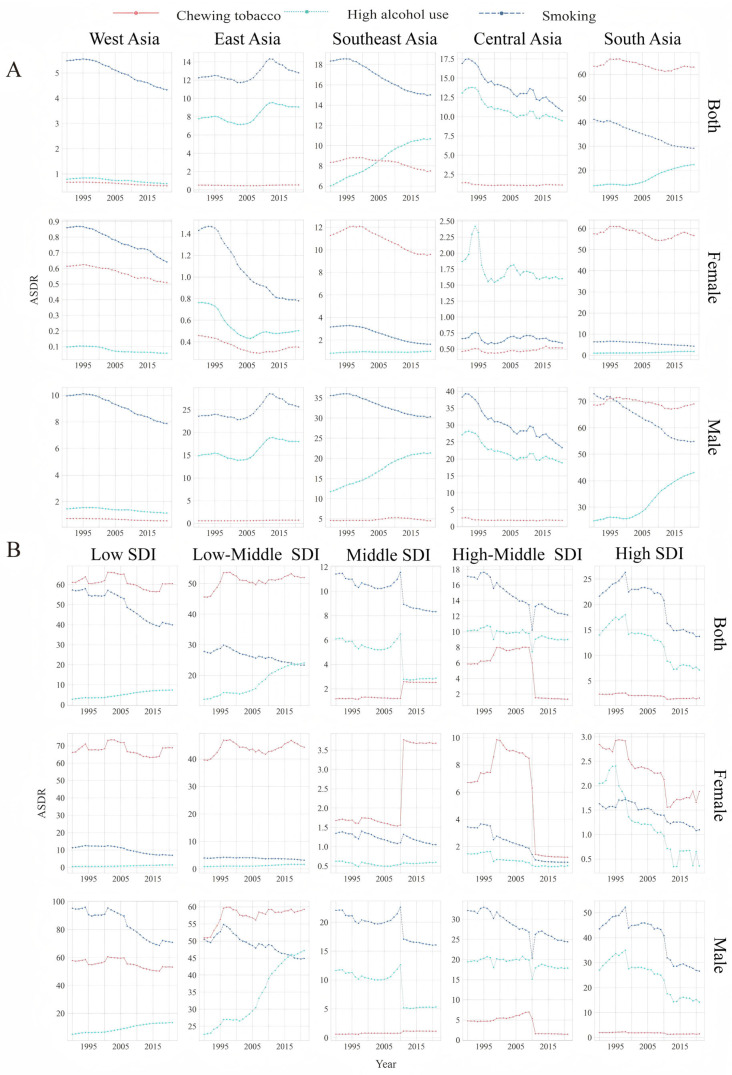
Contribution to the ASDR by risk factors by gender in Asia, 1990–2021. (**A**) Geographical regions. (**B**) SDI regions. SDI: sociodemographic index; DALYs: disability-adjusted life years; ASDR: Age-standardized DALY rate.

Overall, an apparent increasing trend was noted for the ASDR, with an EAPC of 1.03 (95% CI, 1.01 to 1.06) for alcohol-related oral cancer between 1990 and 2021 (Figure 2 and Table 1). In 2021, according to geographic region, South Asia presented the highest ASDR of 22.38 (95% CI, 14.81 to 30.73). This trend was especially evident for males (43.13; 95% CI, 28.65 to 59.42), whose rate that was almost 23-fold greater than that of females (Figure 3A, Table S5). Among the five geographical subregions, the ASDR of Southeast Asia increased the most, with an EAPC of 1.62 (95% CI, 1.60 to 1.63); the same trend was observed in South Asia (1.53 (95% CI, 1.50 to 1.57)) and Southeast Asia (0.61 (95% CI, 0.57 to 0.65)) (Figure 2A and Figure 3A and Table S3).

The ASDRs for alcohol-related oral cancer in the low-SDI and low-middle-SDI regions increased, with EAPCs of 2.71 (95% CI, 2.68 to 2.75) and 2.02 (95% CI, 1.99 to 2.05), respectively (Figure 3B, Table S6). In middle-SDI regions, EAPCs were negative or near zero, while high-middle- and high-SDI regions exhibited further declines. This pattern suggested a shift in the burden of alcohol-related risk from higher to lower SDI regions, with a particularly rapid increase among females with EAPCs of 3.01 (95% CI, 2.98 to 3.04) in low-SDI areas. In 2021, the ASDR for alcohol consumption was highest in the low-middle-SDI regions (47.23; 95% CI, 31.70 to 64.40), especially among males, whose rate was almost 29-fold greater than that among females (1.64; 95% CI, 0.95 to 2.53) and almost eight times greater than that among males in middle-SDI regions (5.34; 95% CI, 3.43 to 7.65) (Figure 3B and Tables S6 and S7).

### 3.3. Trends in the ASDR for LOC Attributable to Tobacco Chewing

The ASDR of LOC attributable to tobacco chewing generally tended to decrease, with an EAPC of −0.04 (95% CI, −0.05 to −0.03) (Figure 2A and Table 1). Among all the regions in Asia, only East Asia had an ASDR that showed an increasing trend, with an EAPC of 0.15 (95% CI, 0.12 to 0.18) (Figure 2A and Table S3) which indicated that the ASDR of oral cancer caused by tobacco chewing was surprisingly high among both males and females in South Asia. In 2021, the ASDR among men in South Asia (69.02; 95% CI, 97.23 to 41.85) was almost 123 times greater than that among men in West Asia (0.56; 95% CI, 0.90 to 0.28), whereas it was almost 111 times greater than that among women (Figure 3A and Table S5). In any case, the only increasing trend in the ASDR was observed in East Asia, for which the EAPC was 0.83 (95% CI, 0.81 to 0.86) (Figure 2A and Table S3).

When tobacco chewing was considered in terms of the SDI, the ASDR increased in the low-middle- and middle-SDI regions, with EAPCs of 0.21 (95% CI, 0.18 to 0.23) and 2.52 (95% CI, 2.41 to 2.64), respectively (Figure 2A and Table S6). The ASDRs in high-SDI regions remained relatively stable. High-middle- and high-SDI regions demonstrated substantial declines for both genders, with females in high–middle-SDI regions experiencing the largest decrease with EAPCs of −5.80 (95% CI, −5.48 to −6.12), suggesting effective control or reduction efforts in these areas. Compared with the other two risk factors, tobacco chewing had the highest disease burden among women in the five SDI regions (Figure 3B and Tables S6 and S7).

### 3.4. Trends in the ASDR for LOC Attributable to Smoking

Smoking is the main cause of oral cancer, which is associated with a high disease burden in Asia. In the exploration of the ASDR for smoking-related oral cancer, a downward trend was observed, with an EAPC of −0.57 (95% CI, −0.56 to −0.58) in Asia (Table 1).

Overall, East Asia was the only geographical region with a slight upward trend in the ASDR, with an EAPC of 0.32 (95% CI, 0.29 to 0.34), and the same trend was observed for men (0.46; 95% CI, 0.43 to 0.48) (Figure 3A and Supplementary Table S3). In contrast, the other four geographical regions and five SDI regions showed decreasing trends, with EAPCs < 0 (Figure 2A,B; Tables S3 and S6). With respect to smoking, the ASDR was relatively high in low-SDI regions, in South Asia, and among males (Figure 3A,B, Tables S5 and S7) in 2021.

With respect to smoking, EAPC values were negative across all SDI regions and genders, indicating an overall downward trend. However, the magnitude of decline varied: high-SDI regions with EAPCs of −1.61 (95% CI, −1.67 to −1.54) had the steepest reductions, while low-middle- and middle-SDI regions showed more modest decreases. In 2021, the ASDR in low-SDI regions (39.93; 95% CI, 23.84 to 59.60) was 4.8 times greater than that in middle-SDI regions (8.33; 95% CI, 5.33 to 11.65) (Figure 3B and Table S4). The ASDR in South Asia (29.19; 95% CI, 18.76 to 39.52) was seven times greater than that in West Asia (4.34; 95% CI, 2.92 to 5.72) and among males (54.85; 95% CI, 35.50 to 74.45) it was 12 times greater than that among females (4.30; 95% CI, 2.57 to 6.49) (Figure 3A and Tables S4 and S6).

### 3.5. Temporal Trends in the ASDR for LOC in Different Age Groups

The ASDR of LOC in Asia increased progressively with age, with a net drift of 0.26 (*p* < 0.05). Notably, the largest increase in the ASDR occurred at ages 20–25 years, and the local drift was 1.02 (Table S8).

**Figure 4:**
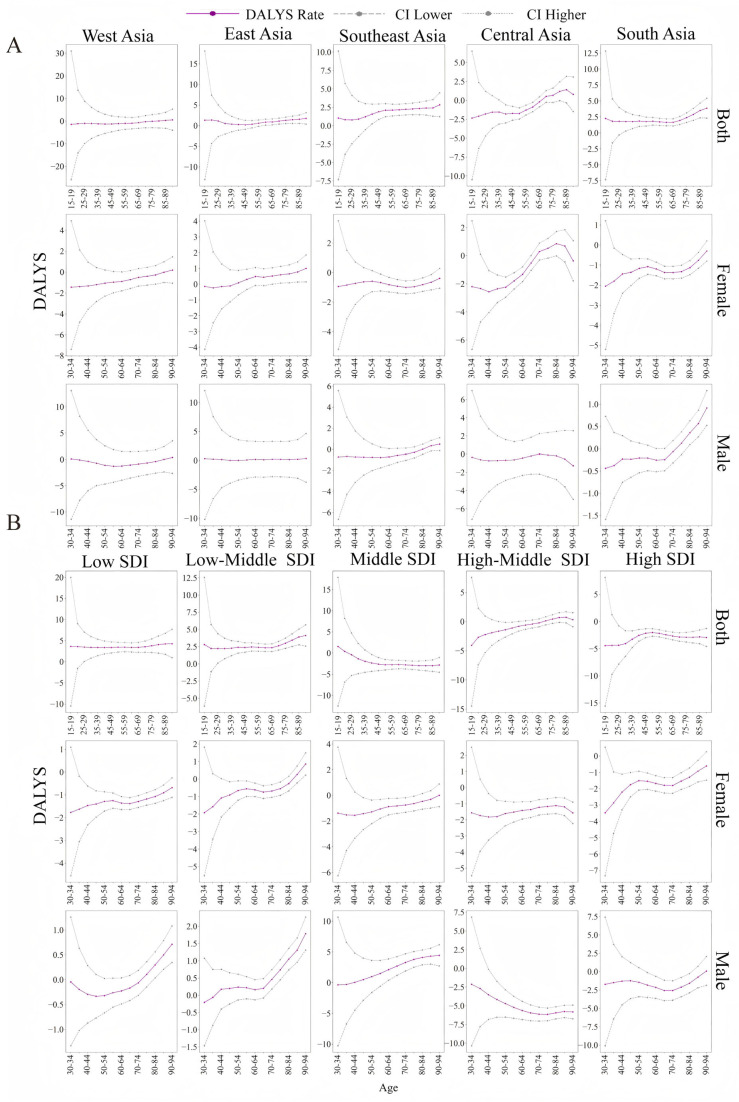
Local drift in LOC attributed to risk factors corresponding with age in geographical and SDI subregions. (**A**) Geographical regions. (**B**) SDI subregions. SDI: sociodemographic index; DALYs: disability-adjusted life years; ASDR: age-standardized DALY rate; CI: confidence interval.

With respect to alcohol consumption, such an increasing trend in the ASDR was found for all age groups in Asia, with a net drift of 1.40 (*p* < 0.05). The ASDRs of Southeast Asia and South Asia also increased, with net drifts of 1.9 and 2.0 (*p* < 0.05), respectively, as did those of the low-SDI and low-middle-SDI regions, with net drifts of 3.5 and 2.6 (*p* < 0.05) respectively (Figure 4A,B and Table 2, Table 3 and Table S8).

As a result of tobacco chewing, the estimated effect of age on the ASDR ebbed and flowed, and only the middle-SDI and low-middle-SDI regions exhibited progressively increasing trends, with net drifts of 2.1 and 0.4, respectively (*p* < 0.05) (Figure 4B and Table 3 and Table S8).

With respect to smoking, the overall trend in the ASDR decreased with age, with a net drift of −0.71 (*p* < 0.05). In addition, Southeast Asia was the only region among the four geographical regions and four SDI regions where the ASDR presented an incremental progression with a net drift of 0.8 (*p* < 0.05) (Figure 4A,B and Table 2, Table 3 and Table S8).

## 4. Discussion

In this study, an updated assessment of the burden of LOC in Asia from 1990–2021 was performed by using GBD 2021 data. This study revealed a spatiotemporal trend of the burden of LOC in Asia according to gender, time, age, geographic region, and economic region. The results revealed that the LOC burden in Asia is still severe and increasing. The disease burden caused by age, gender, smoking, drinking, or tobacco chewing and their effects varies between regions and over time. These findings indicate a persistent disparity in the epidemiology of LOC in Asia, and fine-grained classification and screening of “hot spots” was urgently needed to identify high-risk groups.

### 4.1. Age-Specific Burden Dynamics: Demographic Shifts and Prevention Priorities

Notably, Asia will contain one billion more people by 2050, and half of the population will be older than 65 years old [19]. The APC model revealed that the burden of LOC progressively increases with increasing age, which indicates that the burden of LOC in Asia will present major challenges in the future [20]. Particularly concerning is the vulnerable status of elderly individuals who frequently exhibit inadequate oral health literacy and fail to implement essential self-care practices in their daily routines [20,21]. Compounding these challenges are systemic barriers including limited geriatric healthcare resources, social isolation among solitary-living seniors, and clinical communication difficulties that often manifest through atypical symptom presentation and consequent diagnostic delays [22]. Significantly, our analysis identified the steepest rise in LOC burden among individuals aged 20–25 years, underscoring an urgent public health priority to strengthen prevention initiatives targeting younger populations. This trend aligns with observations by Quan et al. [23] and Pauline et al. [24], who emphasize the critical need for youth-focused interventions. The disproportionate burden arises from insufficient awareness of oral cancer risks [25] and a clinically significant higher recurrence risk compared to older cohorts [26]. These age-specific disparities necessitate tailored policy frameworks, exemplified by China’s Healthy Oral Care Initiative (2019–2025), which adopted a dual approach: prioritizing regionally adapted screening for elderly individuals while integrating youth-oriented prevention programs to address emerging risks. This approach demonstrates how epidemiological insights inform stratified interventions for lifespan-oriented oral health management.

### 4.2. Gender Disparities in LOC Burden

Our findings align with the Global Burden of Disease Study 2019, revealing pronounced gender disparities in the disease burden associated with oral cancer [27], driven by population-specific risk behaviors and sociocultural contexts [12]. Males are disproportionately exposed to occupational hazards such as chemical agents and ultraviolet radiation, compounded by higher rates of smoking and alcohol consumption linked to workplace socialization. Conversely, the traditional stigma around female smoking in many Asian societies has paradoxically normalized smokeless tobacco use among women as a culturally sanctioned alternative [2,12]. These intersecting sociocultural and behavioral factors underscore the necessity for gender-tailored interventions, as advocated by the WHO Framework Convention on Tobacco Control (FCTC). The FCTC prioritizes improving healthcare access for women in smoking-stigmatized cultures while addressing gender-specific risk behaviors, such as alcohol moderation in males and smokeless tobacco reduction in females [28,29].

### 4.3. Risk Factor Heterogeneity: Tobacco, Alcohol, and Smokeless Tobacco Use

Our study revealed that although the disease burden of disease caused by smoking in Asia has decreased, with an EAPC of −0.57 (−0.56, −0.58), the overall ASDR for LOC has remained high. Compared with the other two risk factors, the burden of LOC attributed to smoking was greater in East Asia, West Asia, Southeast Asia, and Central Asia, as well as in the middle-SDI, high-middle-SDI, and high-SDI regions. Tobacco control policies in Asia still need to be strengthened. In terms of the burden of LOC associated with alcohol consumption, three geographic areas (East Asia, Southeast Asia, and South Asia) and two SDI regions (low-SDI and low-middle-SDI regions) had presented increased burdens, with EAPCs > 0. However, smoking and alcohol consumption, which are risk factors for LOC, are prohibited by Islamic law in predominantly Islamic Arab countries, which may explain why West Asia had the lowest ASDR [30]. This may be due to lower awareness of alcohol risks in low-SDI areas, such as insufficient screening initiatives for alcohol dependence [31], and implementation of inadequately effective public health policies, including incomplete bans on alcohol marketing and lack of enforcement [32].

To date, tobacco chewing has been considered one of the leading risk factors for oral cancer [33]. South Asia presents the highest disease burden of disease attributable to tobacco chewing [34], which can be explained by several reasons as follows. First, smokeless tobacco is distributed in the Pacific region, where the environment is suitable for growth, including in South Asia, Southeast Asia, and East Asia [35]. Second, traditional customs and ethnic beliefs about the use of smokeless tobacco might lead to social expectations that strongly encourage people to continue its consumption [36]. Third, South Asia has a deficient primary health care-oriented system [37], and information on smokeless tobacco control policies is inadequate or unavailable in developing countries [2,34]. Additionally, the current regulatory oversight of the ingredients in smokeless tobacco products lags far behind that of cigarettes, which is mainly due to the lack of standardized production and storage methods, high heterogeneity, and the absence of strict laws and policies [38]. Therefore, it is necessary not only to regulate the cultivation, ingredients, and sales of smokeless tobacco but also to strengthen health education efforts at the community level.

Although this study estimates the burden of LOC, it does not fully capture the overall burden of LOC in Asia. The risk factors for LOC in the GBD database are limited, and several potential risk factors and covariates are omitted [39]. In East Asia, unhealthy dietary risk factors, including high salt intake, high preserved and ultra-processed food intake, high sugar-sweetened beverage intake, and low dairy product and whole grain consumption, are positively associated with cancer risk [40]. In addition, smokers tend to drink alcohol, and alcohol drinkers tend to smoke, which results in a synergistic effect between the two risk factors [12]. LOC is caused by the synergistic effect of these two causative factors, which account for three out of every four oral cancer cases worldwide [38]. In East Asia, secondhand smoke may also be strongly associated with oral cancer [41], and thus reducing secondhand smoke may reduce the burden of risk factors in different ways. Finally, since the effect of the coronavirus disease 2019 (COVID-19) pandemic has not been formally incorporated or quantified across a wide range of risk factors or health outcomes, it is likely that COVID-19 accounts for a fraction of the DALYs that would have ensued as a result of other outcomes [42].

### 4.4. Socioeconomic Disparities in LOC Burden: Regional Inequities Across SDI Strata

It is estimated that by 2040, more than two-thirds of the cancer cases worldwide will occur in low- and middle-income countries [43]. This finding is consistent with previous findings of a negative correlation between the oral cancer disease burden and SDI, and an increase in the SDI was conducive to reducing the burden of oral cancer caused by the three risk factors evaluated in this study [43,44]. In this study, tobacco chewing was the largest contributor to the disease burden in low- and low-middle-SDI regions. The reasons are as follows. First, people prefer to use raw materials that are inexpensive. Second, as countries in the lower SDI quintile have insufficient resources for oral health and unsatisfactory dental service systems, patients often lack adequate access to cancer prevention services, timely diagnosis, and various advanced treatments; consequently, these patients eventually miss the optimal window for treatment, which results in low survival rates [2,16]. Finally, poor health awareness might affect timely treatment and intervention [16].

### 4.5. Diagnostic Delays in LOC Burden

Diagnostic delays have been reported to be associated with advanced stage and poor survival outcome in head and neck cancer patients [45]. The delay in diagnosis can be subdivided into three components: patient, professional, and treatment delays [46]. Most patients ignore symptoms and seek care when their cancer is at a later stage (T3 and T4). This is largely due to poor awareness of cancer-related symptoms, as many individuals are unaware that cancer can develop in the oral cavity [5]. Moreover, in less-developed countries, poor healthcare services might lead to later diagnosis and higher mortality rates [47]. In India, approximately 60–80% of patients with oral cancer were diagnosed when their cancer was at an advanced stage, whereas this percentage was 40% in developed countries [48]. Accelerated care pathways and organized screening programs targeting high-risk groups could improve patient survival [49]. Undeniably, we can learn from developed countries regarding the early detection and diagnosis of LOC: for example, the use of AI, which is clinically important for the early diagnosis and screening of LOC in many countries and can help to alleviate the burden on patients and countries [7].

### 4.6. Limitations

An important limitation of our analysis was that data on oral cancer were not complete for all regions and countries. Since the data for West Asia were incomplete, we elected to replace West Asia with North Africa and the Middle East, which have similar geographic environments and customs. In addition to these limitations, the data on lip cancer and oral cancer were combined in the GBD study, which may lead to overestimation [11,50]. The other limitation is that the lack of data on other risk factors, such as betel quid chewing and HIV infection, in the GBD database precludes the ability to quantify the impact of other risk factors on oral cancer.

Finally, while our analysis provides valuable insights into the evolving burden of LOC in Asia, our findings underscore a crucial need for comprehensive, multifaceted cancer control strategies tailored to age, gender, risk factors, and regional contexts. The interplay of economic, social, and cultural factors amplifies the challenges faced by lower SDI regions, where limited resources, underdeveloped healthcare infrastructure, and insufficient public health awareness exacerbate the prevalence of high-risk behaviors such as tobacco chewing. The persistence of such disparities signals an urgent demand for policies that not only address immediate health risks but that also integrate long-term initiatives focused on education, prevention, and early detection. Leveraging technological advances, such as AI-based screening tools, alongside the promotion of culturally sensitive health policies could facilitate early diagnosis, particularly in underserved populations.

## 5. Conclusions

The burden of LOC in Asia continues to increase. Prevention strategies should focus on modifiable risk factors and be tailored to populations of different sexes, ages, and regions, with particular emphasis on men, elderly individuals, lower-SDI regions, South Asia, and East Asia. To address this challenge comprehensively, strengthening primary care systems through routine screening programs and community-based interventions is critical for early detection and risk factor management. The integration of oral health education into public health campaigns is essential to improve awareness of LOC-related risk behaviors. Furthermore, improvement of the healthcare coverage in Asia, especially in high-risk areas, requires multidisciplinary collaboration among oncologists, dentists, public health specialists, and primary care providers to optimize diagnostic pathways, treatment coordination, and survivorship care. To enhance early detection, we recommend implementing risk-stratified screening protocols, such as annual primary care evaluations for populations with elevated LOC risk profiles. Finally, increasing investment in human resources, strengthening infrastructure for cancer care, and establishing region-specific multidisciplinary guidelines are essential to promote the prevention and treatment of LOC.

## Figures and Tables

**Table 1:** ASDR and EAPC in Asia according to geographical region and SDI quintile.

Location Risk	1990	2021	1990–2021
	ASDR (95% UI)	ASDR (95% UI)	EAPC (95% CI)
Global	69.26 (72.95 to −65.92)	67.7141 (73.17 to 61.32)	−0.13 (−0.12 to −0.14)
Asia	77.47 (71.14 to 83.80)	82.23 (73.37 to 90.38)	0.09 (0.08 to 0.10)
**Gender**			
Male	103.08 (92.31 to 114.86)	110.85 (92.68 to 124.52)	0.17 (0.16 to 0.17)
Female	52.14 (46.21 to 57.61)	54.55 (48.39 to 61.07)	−0.04 (−0.02 to −0.05)
Region			
East Asia	32.96 (28.12 to 37.65)	31.88 (25.83 to 38.83)	0.01 (−0.01 to 0.03)
West Asia	19.04 (16.68 to 21.71)	17.74 (15.64 to 20.22)	−0.17 (−0.16 to −0.17)
Cental Asia	53.46 (49.82 to 57.83)	42.26 (36.89 to 48.31)	−0.65 (−0.63 to −0.68)
South Asia	174.46 (156.86 to 192.99)	182.29 (157.06 to 203.68)	0.02 (0.01 to 0.03)
Southeast Asia	63.40 (55.13 to 71.04)	60.97 (69.50 to 52.94)	−0.20 (−0.19 to −0.21)
**SDI level**			
Low SDI	208.84 (257.69 to 162.58)	215.36 (279.07 to 162.27)	−0.10 (−0.08 to −0.13)
Low-middle SDI	123.81 (108.94 to 138.72)	147.51 (125.01 to 167.65)	0.37 (0.35 to 0.39)
Middle SDI	33.64 (27.57 to 39.53)	33.61 (41.60 to 25.96)	0.13 (0.10 to 0.15)
High-middle SDI	55.32 (45.38 to 65.74)	33.00 (25.87 to 41.54)	−1.76 (−1.33 to −1.21)
High SDI	67.07 (57.49 to 78.27)	46.81 (39.27 to 55.72)	−1.27 (−1.21 to −1.33)
**Risk factor**			
Alcohol	9.40 (6.67 to 11.92)	13.26 (9.48 to 17.19)	1.033 (1.005 to 1.06)
Chewing tobacco	19.70 (14.79 to 25.15)	20.29 (15.39 to 24.96)	−0.04 (−0.03 to −0.05)
Smoking	20.94 (15.05 to 26.97)	17.03 (11.61 to 22.48)	−0.57 (−0.56 to −0.58)

Note: UI: uncertainty interval; CI: confidence interval; SDI: sociodemographic index; DALYs: disability-adjusted life years; ASDR: age-standardized DALY rate.

**Table 2:** Local drift with net drift for LOC by geographical regions attributable to each risk factor.

	East Asia			West East			Central Asia			Southeast Asia			South Asia		
	A	C	S	A	C	S	A	C	S	A	C	S	A	C	S
Net Drift	0.8	0.2	0.3	−0.8	−0.7	−0.8 *	−0.9 *	−0.5	−1.1 *	1.9 *	−0.5	0.8 *	2.0 *	−0.1	−1.3 *
Local Drift age group															
15–20	1.3			−1.5			−2.4			1.0			2.3		
20–25	1.4			−1.2			−2.0			0.8			1.8		
25–30	1.1			−1.0			−1.8			0.8			1.8		
30–35	0.5	0.3	−0.1	−1.1	0.1	−1.4	−1.6	−0.4	−2.2	0.9	−0.7	−1.0	1.8	−0.4	−2.1
35–40	0.4	0.2	−0.2	−1.2	−0.1	−1.4	−1.6	−0.6	−2.3	1.2	−0.7	−0.8	1.8	−0.4	−1.8
40–45	0.3	0.2	−0.2	−1.3	−0.4	−1.3	−1.8	−0.7	−2.6	1.6	−0.7	−0.7	1.9	−0.2	−1.4
45–50	0.2	0.02	−0.1	−1.3	−0.7	−1.2	−1.7	−0.7	−2.7	1.9	−0.8	−0.6	1.8	−0.2	−1.4
50–55	0.3	0.02	0.1	−1.1	−1.1	−1.1	−1.7	−0.7	−2.2	2.1	−0.8	−0.6	1.8	−0.2	−1.2
55–60	0.6	0.1	0.3	−1.0	−1.3	−1.0	−1.3	−0.6	−1.8	2.1	−0.8	−0.7	1.7	−0.2	−1.1
60–65	0.8	0.2	0.5	−1.0	−1.3	−0.9	−0.9	−0.4	−1.3	2.2	−0.7	−0.8	1.7	−0.3	−1.2
65–70	0.9	0.1	0.4	−0.7	−1.1	−0.7	−0.2	−0.2	−0.5	2.2	−0.6	−0.9	1.7	−0.3	−1.4
70–75	1.1	0.2	0.5	−0.3	−0.9	−0.5	0.5	0.0	0.3	2.3	−0.5	−1.0	1.9	−0.1	−1.4
75–80	1.3	0.2	0.6	−0.1	−0.7	−0.4	0.7	−0.1	0.5	2.3	−0.3	−1.0	2.4	0.1	−1.3
80–85	1.4	0.2	0.7	0.04	−0.4	−0.3	1.2	−0.2	0.9	2.4	0.0	−0.8	2.9	0.4	−1.1
85–90	1.6	0.2	0.8	0.3	−0.0	−0.0	1.4	−0.6	0.7	2.4	0.3	−0.7	3.5	0.6	−0.8
90–95	1.7	0.4	1.0	0.5	0.4	0.2	0.8	−1.3	−0.4	2.8	0.5	−0.4	3.9	0.9	−0.3

Note: * *p* < 0.05; LOC,: lip and oral cavity cancer. A: alcohol consumption; C: chewing tobacco; S: smoking.

**Table 3:** Local drift with net drift for cancer by SDI regions attributable to each risk factor.

	High SDI			High-Middle SDI			Middle SDI			Low-Middle SDI			Low SDI		
	A	C	S	A	C	S	A	C	S	A	C	S	A	C	S
Net Drift	−2.9 *	−1.7 *	−1.7 *	−0.9 *	−5.1 *	−1.5 *	−2.3	2.1 *	−0.9 *	2.6 *	0.4 *	−0.7 *	3.5 *	−0.1	−1.3 *
Local Drift age group															
15–20	−4.5			−4.1			1.5			2.8			3.6		
20–25	−4.4			−2.7			0.4			2.2			3.6		
25–30	−4.4			−2.2			−0.4			2.2			3.5		
30–35	−4.1	−1.7	−3.5	−1.9	−2.1	−1.6	−1.4	−0.4	−1.4	2.2	−0.2	−1.9	3.4	−0.0	−1.8
35–40	−3.3	−1.5	−2.9	−1.7	−2.7	−1.7	−2.0	−0.3	−1.5	2.3	−0.1	−1.6	3.4	−0.2	−1.6
40–45	−2.6	−1.3	−2.2	−1.4	−3.5	−1.8	−2.4	0.1	−1.6	2.4	0.2	−1.1	3.4	−0.3	−1.5
45–50	−2.2	−1.2	−1.8	−1.1	−4.2	−1.8	−2.6	0.5	−1.4	2.4	0.2	−0.9	3.4	−0.3	−1.4
50–55	−2.0	−1.5	−1.5	−0.8	−4.7	−1.6	−2.8	1.0	−1.3	2.5	0.2	−0.7	3.4	−0.3	−1.3
55–60	−2.2	−1.8	−1.6	−0.6	−5.2	−1.5	−2.8	1.5	−1.1	2.4	0.2	−0.6	3.5	−0.3	−1.3
60–65	−2.4	−2.2	−1.7	−0.4	−5.6	−1.4	−2.7	2.1	−0.9	2.3	0.2	−0.6	3.4	−0.2	−1.4
65–70	−2.7	−2.6	−1.8	−0.2	−6.0	−1.4	−2.8	2.7	−0.8	2.3	0.2	−0.8	3.4	−0.2	−1.4
70–75	−2.9	−2.6	−1.8	0.1	−6.1	−1.2	−2.9	3.3	−0.8	2.6	0.5	−0.7	3.6	−0.1	−1.3
75–80	−2.9	−2.1	−1.5	0.4	−6.1	−1.2	−3.0	3.8	−0.6	3.0	0.7	−0.6	3.8	0.1	−1.2
80–85	−2.9	−1.6	−1.3	0.7	−5.9	−1.1	−3.0	4.1	−0.5	3.4	1.1	−0.3	4.1	0.3	−1.1
85–90	−2.8	−0.7	−0.9	0.7	−5.8	−1.2	−3.0	4.3	−0.3	3.9	1.3	0.3	4.2	0.5	−0.9
90–95	−3.0	0.1	−0.6	0.3	−5.8	−1.6	−2.8	4.4	−0.0	4.1	1.8	0.9	4.3	0.7	−0.7

Note: * *p* < 0.05; A: alcohol consumption; C: chewing tobacco; S: smoking.

## Data Availability

Data are contained with in the article.

## Supplementary Materials

The following supporting information can be downloaded at: https://www.mdpi.com/article/10.3390/healthcare13121377/s1, Table S1: The DAILYs of Global and Asian for LOC in 1990, 2019, 2021; Table S2: ASDR in different geographic regions by gender from 1990 to 2021; Table S3: EAPC of different regions and genders with different risk factors; Table S4: ASDR for different SDI regions from 1990 to 2021; Table S5: The DAILYs of Asian LOC for different risk factors in different geographic regions from 1990 to 2021; Table S6: EAPC of different risk factor and genders in different SDI regions; Table S7: ASDR in different SDI regions with different risk factors and genders; Table S8: Local Drift and Net Drift for LOC attributable to each risk factor and genders in Asia; Table S9: The deaths and DAILYs rate on different risk factors by gender from 1990 to 2021; Table S10: List of Asian countries by geographic region; Table S11: SDI for different years in different Asian countries.

## Abbreviations

The following abbreviations are used in this manuscript:

LOC	lip and oral cancers
SDI	sociodemographic Index
GBD	global burden of disease
DALYs	disability-adjusted life years
ASDR	age-standardized DALY rate
EAPC	estimated annual percentage change
