# Supplementary material for: Spatiotemporal Dynamics in the Burden of Lip and Oral Cavity Cancer and Attributable Risk Factors in Asia (1990–2021)
Source: Healthcare (Basel). 2025 Jun 9;13(12):1377. doi: 10.3390/healthcare13121377 (PMC12193132; doi:10.3390/healthcare13121377)
Supplement: Supplementary file 1 [file healthcare-13-01377-s001.zip › Table S1. The DAILYs of Global and Asian for LOC in 1990, 2019, 2021..pdf]

Table S1. The DAILYs of Global and Asian for LOC in 1990, 2019, 2021.

Region	The Number of DAILYs		
	1990	2019	2021
Asia	1781211.87	4082200.38	4283251.95
Global	2936205.46	5664617.17	5874069.63

DALYs: disability-adjusted life years; LOC: lip and oral cancers.
