# Supplementary material for: Spatiotemporal Dynamics in the Burden of Lip and Oral Cavity Cancer and Attributable Risk Factors in Asia (1990–2021)
Source: Healthcare (Basel). 2025 Jun 9;13(12):1377. doi: 10.3390/healthcare13121377 (PMC12193132; doi:10.3390/healthcare13121377)
Supplement: Supplementary file 1 [file healthcare-13-01377-s001.zip › Table S10. List of Asian countries by geographic region.pdf]

Table S10. List of Asian countries by geographic region.

Region	Countries
East Asia	Democratic People's Republic of Korea
	Mongolia
	People's Republic of China
	Taiwan (Province of China)
	Japan
Southeast Asia	Kingdom of Cambodia
	Republic of Indonesia
	Lao People's Democratic Republic
	Malaysia
	Republic of Maldives
	Republic of Mauritius
	Republic of the Union of Myanmar
	Republic of the Philippines
	Republic of Seychelles
	Democratic Socialist Republic of Sri Lanka
	Kingdom of Thailand
	Democratic Republic of Timor-Leste
	Viet Nam
South Asia	Republic of India
	Islamic Republic of Afghanistan
	People's Republic of Bangladesh
	Kingdom of Bhutan
	Federal Democratic Republic of Nepal
	Islamic Republic of Pakistan
Central Asia	Republic of Armenia
	Republic of Azerbaijan
	Georgia
	Republic of Kazakhstan
	Kyrgyz Republic
	Republic of Tajikistan
	Turkmenistan
	Republic of Uzbekistan
West Asia	People's Democratic Republic of Algeria
	Kingdom of Bahrain
	Arab Republic of Egypt
	Islamic Republic of Iran
	Republic of Iraq
	Hashemite Kingdom of Jordan
	State of Kuwait
	Lebanese Republic
	State of Libya
	Kingdom of Morocco
	Sultanate of Oman

	Palestine
	State of Qatar
	Kingdom of Saudi Arabia
	Republic of Sudan
	Republic of Tunisia
	Republic of Turkey
	Syrian Arab Republic
	Republic of Yemen
