# Supplementary material for: Spatiotemporal Dynamics in the Burden of Lip and Oral Cavity Cancer and Attributable Risk Factors in Asia (1990–2021)
Source: Healthcare (Basel). 2025 Jun 9;13(12):1377. doi: 10.3390/healthcare13121377 (PMC12193132; doi:10.3390/healthcare13121377)
Supplement: Supplementary file 1 [file healthcare-13-01377-s001.zip › Table S8. Local Drift and Net Drift for LOC attributable to each risk factor and genders in Asia.pdf]

**Table S8.** Local Drift and Net Drift for LOC attributable to each risk factor and genders in Asia.

| | | Asia(risk factors) | | | Asia(sex) | | |
|---|---|---|---|---|---|---|---|
| | | A | C | S | Both | M | F |
| Net Drift | | 1.40* | 0.021 | −0.71* | 0.26* | 0.34* | 0.12 |
| Local Drift | age group | | | | | | |
| | 15−20 | 2.08 | | | 0.87 | 0.83 | 0.88 |
| | 20−25 | 2.00 | | | 1.02 | 0.99 | 1.06 |
| | 25−30 | 1.86 | | | 0.91 | 0.94 | 0.85 |
| | 30−35 | 1.54 | 0.47 | −1.20 | 0.91 | 0.71 | 0.59 |
| | 35−40 | 1.49 | 0.51 | −1.05 | 0.65 | 0.72 | 0.55 |
| | 40−45 | 1.37 | 0.34 | −0.87 | 0.45 | 0.58 | 0.25 |
| | 45−50 | 1.10 | −0.14 | −0.93 | 0.04 | 0.21 | −0.23 |
| | 50−55 | 1.06 | −0.30 | −0.81 | −0.10 | 0.11 | −0.45 |
| | 55−60 | 1.08 | −0.23 | −0.69 | −0.08 | 0.09 | −0.34 |
| | 60−65 | 1.12 | −0.13 | −0.66 | −0.06 | 0.07 | −0.27 |
| | 65−70 | 1.11 | −0.09 | −0.72 | −0.10 | −0.04 | −0.22 |
| | 70−75 | 1.27 | 0.13 | −0.64 | 0.05 | 0.02 | 0.03 |
| | 75−80 | 1.38 | 0.22 | −0.56 | 0.16 | 0.09 | 0.21 |
| | 80−85 | 1.52 | 0.02 | −0.56 | 0.17 | 0.15 | 0.20 |
| | 85−90 | 1.86 | −0.12 | −0.42 | 0.28 | 0.40 | 0.22 |
| | 90−95 | 2.36 | −0.06 | −0.16 | 0.54 | 0.70 | 0.47 |

LOC: lip and oral cancers; A: alcohol consumption; C:chewing tobacco; S: smoking; M: males; F: females;both： male and female; * $p < 0.05$.
